# Antiarrhythmic drug therapy among patients presenting to emergency department with symptomatic atrial fibrillation – a prospective nationwide cohort

**DOI:** 10.1186/s13049-017-0424-7

**Published:** 2017-08-15

**Authors:** Tero Penttilä, Heikki Mäkynen, Juha Hartikainen, Harri Hyppölä, Timo Lauri, Mika Lehto, Juha Lund, MJ Pekka Raatikainen, for the FinFib2 investigators, Pekka Raatikainen, Mika Lehto, Aleksi Almenoksa, Juha Koskinen, Sergei Kesonen, Jyri Veräjänkorva, Kimmo Salmio, Juhani Metsäniitty, Laura Mikkonen, Jouni Nurmi, Risto Viitanen, Jukka Vaahersalo, Jukka Rinne, Risto Pajari, Jani Mononen, Anna-Mari Hekkala, Laura Moring, Bernd Günther, Juha Lund, Jarkko Karihuhta, Jon Holmström, Juho Lindberg, Anja Toljamo, Jonna Juhola, Marjatta Strandberg, Tuula Meinander, Heikki Mäkynen, Tero Penttilä, Ville Hällberg, Tapio Innamaa, Hanna Suurmunne, Jari Nyrhilä, Katja Jokela, Peeter Kasemets, Juha Hartikainen, Matti Onnela, Harri Hyppölä, Kai Nyman, Pirjo Mustonen, Tuomas Rissanen, Jaana Luukkonen, Pertti Salmi, Pekka Salminen, Teemu Lasanen, Matti Kettunen, Timo Lauri, Ari Toppinen, Jussi Sia, Hanna Tormilainen, Magnus Hagnäs, Tapio Åman, Liisa Miettinen, Niilo Keränen

**Affiliations:** 1 Heart Center Co. Tampere University Hospital, P.O. Box 2000, -33521 Tampere, FI Finland; 2 Heart Center, Kuopio University Hospital, Kuopio, Finland; 3 Emergency Department, Kuopio University Hospital, Kuopio, Finland; 4 Oulu University Hospital, Oulu, Finland; 5 Heart and Lung Center, Helsinki University Hospital, Helsinki, Finland; 6 Turku University Hospital, Turku, Finland

**Keywords:** Atrial fibrillation, Emergency department, Antiarrhythmic medication, Rhythm control, Rate control, EHRA score

## Abstract

**Background:**

Atrial fibrillation (AF) is a common arrhythmia that causes numerous visits to emergency departments (ED). The aim of the FinFib2 study was to evaluate whether treatment of patients with AF in ED is consistent with the contemporary European Society of Cardiology (ESC) management guidelines. Here we report the results of antiarrhythmic drug therapy (AAD) in ED.

**Methods:**

All patients within the two-week study period whose primary reason for the ED visit was symptomatic AF were included into this prospective multicentre study. Comprehensive data on factors contributing to the treatment of AF were collected, including a data of previous use of ADDs, and changes made for them during a visit in ED.

**Results:**

The study population consisted of 1013 consecutive patients (mean age 70 ± 13 years, 47.6% female). The mean European Heart Rhythm Association (EHRA) symptom score was 2.2 ± 0.8. Rhythm control strategy was opt for 498 (63.8%) and 140 (64.5%) patients with previously and newly diagnosed AF, respectively. In patients with previously diagnosed AF the most frequently used AAD was a beta blocker (80.9%). Prior use of class I (11.4%) and III (9.1%) AADs as well as start or adjustment of their dosage (7.4%) were uncommon. Most of the patients with newly diagnosed AF were prescribed a beta blocker (71.0%) or a calcium channel antagonist (24.0%), and only two of them received class I or class III AADs.

**Conclusions:**

Our data demonstrated that in patients presenting to the ED with recurrent symptomatic AF and aimed for rhythm control strategy, the use of class I and class III AADs was rare despite ESC guideline recommendations. It is possible that early adaptation of a more aggressive rhythm control strategy might improve a quality of life for symptomatic patients and alleviate the ED burden associated with AF. Beta blockers were used by majority of patients as rate control therapy both in rate and rhythm control groups.

**Trial registration:**

NCT01990105. Registered 15 November 2013.

## Background

Atrial fibrillation (AF) is the most common sustained arrhythmia [1]. Given the predicted increase in the incidence of AF it has been estimated that in 2030 there will be 14–17 million patients with AF in Europe [2–4]. Consequently, the amount of AF related visits to the emergency departments (ED) is likely to rise extensively in near future [5].

Emergency departments play a key role in management of AF [6]. In patients with acute AF the decision between rhythm and rate control is done in the ED. The severity of symptoms related to AF is the main factor in selection of the treatment strategy [7]. Age, presence of structural heart disease and other co-morbidities, the type of AF and contraindications to antiarrhythmic drugs (AAD) should also be taken into account when evaluating the need and reasonability of cardioversion, and how to prevent recurrent AF episodes [8]. The efficacy and safety of beta blockers and calcium channel antagonists are well established in acute and long-term rate control [9]. Rate control therapy should consider for all patients with AF if needed, both in a rate and a rhythm control strategies [10, 11]. In a rhythm control strategy, additionally class I and III AADs are recommended to maintain sinus rhythm [12–17]. Regardless of the chosen treatment strategy the need of oral anticoagulation (OAC) along with risk factors for thromboembolic complications and bleeding must be evaluated [10, 11].

The results of previous studies indicate that there is large variation in the management of AF in the ED [18, 19]. The FinFib2 study was designed to evaluate whether the treatment of patients with symptomatic AF in the ED is in line with the European Society of Cardiology (ESC) treatment guidelines valid at the study period 2013 [10, 11]. We report real life data on the selection of the treatment strategy, symptoms and risk factors of AF, and use of antiarrhythmic medication in these patients.

## Methods

### Study design and patient population

This prospective snapshot study was conducted in 35 EDs around Finland. There were a large variation of size and facilities of EDs; units from small heath care centers to big university hospitals were participating. Finland is divided into five university hospital districts, and patients from all of them were enrolled in order to avoid any bias due to geographical differences. All patients whose primary reason for the ED visit was symptomatic AF during a two-week study period (November 11–23, 2013) were included.

**Fig. 1:**
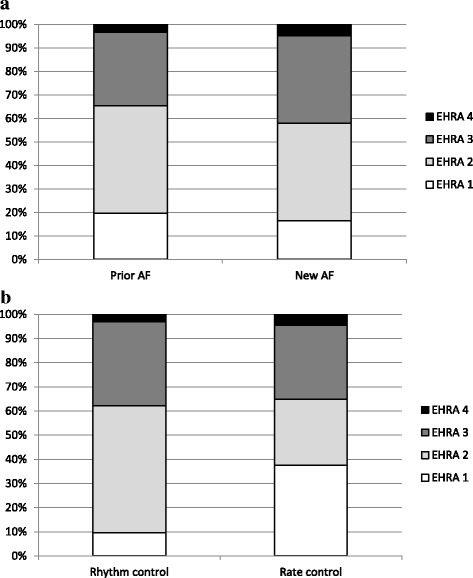
Classification of AF related symptoms according to the European Heart Rhythm Association (EHRA) score in patients with prior (*n* = 780) and newly diagnosed AF (*n* = 217) (**a**) and in patients with rhythm control (*n* = 659) and rate control strategy (*n* = 336) (**b**). EHRA 1 = no symptoms, EHRA 2 = mild symptoms (normal daily activity not affected), EHRA 3 = severe symptoms (normal daily activity affected), EHRA IV = disabling symptoms (normal daily activity discontinued)

Data on concomitant diseases, risk factors for AF, and a history of thromboembolic complications, treatment strategy, and use of antiarrhythmic medication was collected using predefined internet based case report form. A rhythm control strategy means a physician’s aim to maintain a sinus rhythm. A rate control strategy means a physician’s decision to accept a permanent AF. Symptoms associated to AF were ranked using European Heart Rhythm Association (EHRA) score [10] (Fig. 1). Antiarrhythmic drugs were classified according to the Vaughan Williams classification (Table 1).

**Table 1:** Antiarrhythmic drugs according to the Vaughan Williams classification. None of the patients received drugs which are presented in parenthesis

Classification	Agents	Mechanism of action	Notes
IA	(Disopyramide) Quinidine (Procainamide)	Sodium channel blockade with intermediate association/dissociation and potassium channel blockade	Contraindicated in patients with structural heart diseases
IB	(Lidocaine) (Mexiletine)	Sodium channel blockade with rapid association/dissociation	Not indicated for AF
IC	Flecainide (Propafenone)	Sodium channel blockade with slow association/dissociation	Contraindicated in patients with structural heart diseases
II	(Atenolol) (Asebutolol) (Betaxolol) Bisoprolol Carvedilol Metoprolol (Nebivolol) (Pindolol) Propranolol (Seliprolol)	Beta adrenergic receptor blockade	Can be used also in patients with structural heart disease More effective in rate than rhythm control
III	Amiodarone Dronedarone Sotalol Vernakalant	Potassium channel blockade Amiodarone and dronedarone have also class I, II and IV activity Sotalol has also class II activity Vernakalant blocks sodium and potassium channels in atria but not in ventricles	Dronedarone is contraindicated in severe heart failure and permanent AF Extra cardiac adverse events (e.g., liver and pulmonary toxicity and thyroid dysfunction) are common with amiodarone Vernakalant is available only for acute intravenous use
IV	Verapamil Diltiazem	Calcium channel blockade	Should be avoided in patients with congestive heart failure
Others	Digoxin	Variable mechanisms	May have adverse effect on the prognosis of patients with AF

A data of prior use and changes made for AADs in ED were collected. A physician in ED made an independent decision to choose a rate control strategy or a rhythm control strategy for each patient. We evaluated the use of AAD therapy during an ED visit to support that decision; if the rhythm control was chosen, were a class I or III AAD started or a dosage of these drugs changed. We also evaluated a use of rate control drugs (beta and calcium channel blockers) for all patients, both in a rate and a rhythm control groups.

### Statistical analysis

The data were analysed using IBM SPSS Statistics software package version 22 (IBM SPSS Inc., Armonk, NY, USA). Missing data values were excluded from the statistical analysis. Continuous variables are expressed as mean ± standard deviation and compared with independent variables t-test or Mann-Whitney U-test when appropriate. Categorical variables are expressed as numbers and percentages and compared by Fisher’s exact test. All tests were two-sided and a *P* value of <0.05 was considered statistically significant.

## Results

### Baseline characteristic of the study population

A total of 1013 consecutive patients with symptomatic AF were enrolled into the study. In 217 (21.4%) patients AF was diagnosed for the first time in the ED, whereas 780 (77.0%) patients had recurrent AF. The mean age of the patients was 70.0 ± 13.1 years (19–103 years) - 67.7% of them were 65 years or older, and 39.5% were 75 years or older. Slightly less than half of them (47.6%) were female. The most common underlying diseases were hypertension (65.0%), dyslipidaemia (43.5%), coronary artery disease (23.2%), and diabetes (21.1%) (Table 2).

**Table 2:** Clinical characteristics of the patients with symptomatic atrial fibrillation (AF) on admission to the emergency department in the rhythm versus rate control groups

	Total n (%)	Rhythm control n (%)	Rate control n (%)	*P*-value
Patients	1013	659 (65.1)	336 (33.2)	
Previously diagnosed AF	780	498 (63.8)	261 (33.5)	
Newly diagnosed AF	217	140 (64.5)	71 (32.7)	
Age	70.0 ± 13.1 (19–103)	65.7 ± 12.4	78.6 ± 10.1	< 0.001
Female	482 (47.6)	267 (40.5)	205 (61.0)	< 0.001
Congestive heart failure	176 (17.4)	57 (8.6)	115 (34.2)	< 0.001
Hypertension	658 (65.0)	395 (59.9)	251 (74.7)	< 0.001
Diabetes	214 (21.1)	116 (17.6)	95 (28.3)	< 0.001
Stroke	81 (8.0)	37 (5.6)	41 (12.2)	< 0.001
Transient ischemic attack	53 (5.2)	21 (3.2)	32 (9.5)	< 0.001
Other thromboembolic events	24 (2.4)	8 (1.2)	15 (4.5)	0.003
Coronary artery disease	235 (23.2)	106 (16.1)	122 (36.3)	< 0.001
Previous myocardial infarction	126 (12.4)	55 (8.3)	66 (19.6)	< 0.001
Atherosclerosis	39 (3.8))	15 (2.3)	24 (7.1)	< 0.001
Dyslipidaemia	441 (43.5)	273 (41.4)	161 (47.9)	0.042
Ongoing or ex-smoking	254 (25.1)	174 (26.4)	75 (22.3)	0.809
Valvular disease	128 (12.6)	63 (9.6)	65 (19.3)	< 0.001
Thyroid dysfunction	112 (11.1)	66 (10.0)	45 (13.4)	0.136
Lung disease	135 (13.3)	72 (10.9)	61 (18.2)	0.002
Renal insufficiency	100 (9.9)	27 (4.1)	70 (20.8)	< 0.001
Liver insufficiency	18 (1.8)	7 (1.1)	11 (3.3)	0.021
Anaemia	103 (10.2)	39 (5.9)	62 (18.5)	< 0.001
History of major bleeding	34 (3.4)	16 (2.4)	18 (5.4)	0.026
Echocardiography	508 (50.1)	353 (53.6)	152 (45.2)	0.015
EHRA score	2.2 ± 0.8	2.3 ± 0.7	2.0 ± 0.9	< 0.001
CHA2DS2-VASc score	3.1 ± 2.1	2.4 ± 1.9	4.4 ± 1.8	< 0.001
HAS-BLED score	1.9 ± 1.2	1.5 ± 1.1	2.6 ± 1.2	< 0.001

The mean EHRA score was 2.2 ± 0.8. The most frequent symptom was palpitation (620 patients, 61.2%). Other AF related symptoms included dyspnoea (270 patients, 26.7%), dizziness (197 patients, 19.4%), and chest pain (125 patients, 12.3%). Eighteen patients (1.8%) had had syncope. Almost half of the patients with previously diagnosed AF (354 patients, 45.4%) had had at least one visit to the ED because of AF within the preceding 12 months. For these patients, the mean number of prior ED admissions per patient was 2.7 ± 3.6 (range 1–30).

### Prior antiarrhythmic therapy

Most of the patients (85.3%) with previously diagnosed AF were using antiarrhythmic medication for rhythm or rate control when admitted to the ED. Beta blockers were used by 631 (80.9%), digoxin by 72 (9.2%), and verapamil or diltiazem by 23 (2.9%) of these patients. Class I AADs were used by 89 (11.4%) patients. The majority of them were using flecainide (87 patients, 11.2%), and two patients were on quinidine (0.3%). The other AADs included amiodarone (26 patients, 3.3%), dronedarone (30 patients, 3.8%) and sotalol (15 patients, 1.9%).

### Treatment strategy and initial management in the ED

In patients with previously diagnosed AF the rhythm disorder was paroxysmal in 282 (36.5%), persistent in 298 (38.7%) and permanent in 191 (24.8%) patients. Rhythm control strategy was chosen by ED physicians in 498 of the 580 (85.9%) patients with paroxysmal or persistent AF. Among them 287 and 62 patients underwent successful acute electrical or pharmacological cardioversion, respectively. Sinus rhythm restored spontaneously or an elective cardioversion were planned in 149 patients. The AADs used for pharmacological cardioversion included flecainide (17 patients), amiodarone (7 patients), vernakalant (6 patients), and a beta blocker or digoxin (32 patients).

There were 217 patients with newly diagnosed AF in the study population. The rhythm control strategy was chosen for 140 (64.5%) of them. Normal sinus rhythm resumed spontaneously in 51 patients. Electrical or pharmacological cardioversion was performed in 43 and 21 (beta blocker 17, digoxin 1, flecainide 3, amiodarone 1) patients, respectively. An elective cardioversion was planned for 25 patients.

The patients in whom rate control strategy was chosen were significantly older than those with rhythm control strategy (78.6 ± 10.1 vs. 65.7 ± 12.4 years, *P* < 0.001). The EHRA score was significantly higher among patients with rhythm control strategy (2.3 ± 0.7 vs. 2.0 ± 0.9, *P* < 0.001), whereas the CHA_2_DS_2_VASc (4.4 ± 1.8 vs. 2.4 ± 1.9, *P* < 0.001) and HAS-BLED (2.6 ± 1.2 vs. 1.5 ± 1.1, *P* < 0.001) scores were higher among those with rate control strategy (Table 2).

### Antiarrhythmic drug therapy at discharge

At discharge from the ED 625 (80.1%) patients with prior AF diagnosis were prescribed beta blocker medication. Despite recurrent AF symptoms a change in antiarrhythmic medication was done only in 97 of these patients (12.4%). Class I or III antiarrhythmic medication was started or the dosage of the drug used was adjusted in 37 (7.4%) of the patients with previous AF diagnosis and rhythm control strategy. The reasons for the changes in AAD therapy included inadequate antiarrhythmic efficacy (68 patients), adverse effects (11 patients), change into rate control strategy (19 patients) and lack of compliance (1 patient).

In patients with newly diagnosed AF a beta blocker was prescribed for 154 (71.0%), a calcium channel antagonist for 52 patients (24.0%), and digoxin for 8 patients (3.7%). Amiodarone was started for 2 patients, but no class I drugs were prescribed for the patients with newly diagnosed AF.

## Discussion

**Fig. 2:**
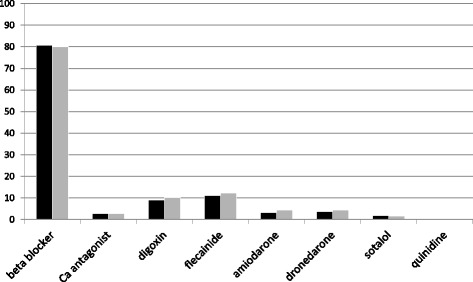
The use of antiarrhythmic drugs among patients with previously diagnosed AF (*n* = 780) at admission to the ED (*black column*) and at discharge (*light grey column*)

The results of the FinFib2 study demonstrated that despite recurrent symptoms and ESC guideline recommendations prior use and initiation of class I and class III antiarrhythmic medication are rare in patients presenting to the ED with symptomatic AF (Fig. 2).

At admission to the ED class I or III antiarrhythmic drugs were used only by 20.5% of the patients with prior AF diagnosis, and at discharge 36.5% of the patients in the rhythm control group were prescribed class I or III antiarrhythmic medication. With regard to the current guidelines recommending execution of “upstream” therapy and use of class I and III antiarrhythmic drugs in the early stage of the disease, these numbers appear low [20, 21]. In patients with highly symptomatic AF active rhythm control strategy is expected not only to improve the quality of life, but also to reduce ED burden associated with AF.

After cardioversion, the likelihood of AF recurrences is highest during the following weeks [22]. It has been shown that 56% of the patients presenting to the ED with symptomatic AF will have a repeat ED visit due to AF recurrence within a year [18]. This was reflected in our study population by the high number of previous visits to ED. In patients with acute AF normal sinus rhythm can be effectively restored by electrical or pharmacological cardioversion [20]. However, in rhythm control strategy cardioversion alone is hardly ever enough but a physician should always try to find a way to prevent AF recurrences. In our survey, a class I or III antiarrhythmic medication was started or the dosage was optimized as a part of a rhythm control strategy only in about 7% of the patients with a previously diagnosed AF. It is well established that early intervention is crucial for the long-term efficacy of antiarrhythmic drugs and catheter ablation. Therefore, decision to start a class I or III antiarrhythmic medication should be done in the ED if structural heart disease has been excluded by recent examinations and clinical history. Echocardiography plays a key role in diagnosis of many cardiac diseases and selection of AF treatment strategy. In our study, it had been done earlier for half of the patients.

However, as the rhythm control strategy has not demonstrated a mortality benefit compared to the rate control strategy in large studies, it should be not used for all patients with AF [23–25]. In our study, patients in the rate control group were older, had more concomitant diseases, and a lower symptom score.

A rate control therapy should be considered for all patients with AF despite of a treatment strategy, if a heart rate during AF is rapid. In this study population, a rate control therapy was used by most of the patients. In line with the results of previous studies, about 80% of the patients in our study were using beta blockers [26]. The efficacy of beta blockers to reduce ventricular rate in patients with AF is well established, but their efficacy in preventing AF recurrences is only modest [22, 27].

According to the contemporary AF management guidelines class I agents should be the drug of choice for rhythm control in patients with lone AF. Despite this, significantly less effective beta blocker medication was used as a first line therapy in most of these patients, and none of them received class I antiarrhythmic medication after a first documented AF episode.

The facilities to treat patients with AF vary between different EDs in Finland. In small heath care centers it’s not possible to make an electrical cardioversion, and a patient has to be sent to a bigger unit. In smaller EDs physicians experience to use AADs might not be as good as in bigger units. In Finland, we are going towards bigger ED units in near future, which is expected to improve the quality of rhythm control therapy of patients with AF, and also improve acute collaboration between ED physicians and cardiologists.

## Limitations

This study provided a snapshot of the management of patient with AF in an emergency care setting. Therefore, no follow-up data are available. For example, many patients were referred to a cardiologist but the results of the further examinations and therapies were not available. Likewise, it was not possible to assess a rationality of all individual treatment decisions; therefore we assessed a treatment decisions the physicians made after choosing a treatment strategy. Finally, the results of this study may not be directly extrapolated to different health care systems. In Finland all inhabitants are covered by national health insurance and patients need to pay only a nominal fee for ED visits and hospitalization.

## Clinical implications

ED physicians play a key role in treatment of acute AF. We have previously shown that patients with high risk of thromboembolic complications were recognized well by the ED physicians, and oral anticoagulation therapy (OAC) was prescribed for the majority of these patients [28]. The results of the current analysis indicate that rhythm control therapy is not understood and executed as well as OAC therapy. According to contemporary ESC clinical practice guidelines rhythm control strategy should be considered for patients with recurrent episodes of symptomatic AF [10, 11]. On the other hand, rate control strategy is a feasible choice in patients with mild symptoms. In many cases the treatment strategy can be selected and implemented in the ED. In our study the factors favouring a rate control strategy included older age, concomitant diseases and mild/moderate symptoms.

Early adaptation of an aggressive rhythm control strategy is likely to alleviate the ED burden associated with symptomatic AF. In order to be able to select the most appropriate antiarrhythmic therapy for a given patient the ED physician should evaluate the symptoms of the patient and take thorough clinical history. He/she should have enough information on the results of prior cardiac examinations (e.g., electronic nationwide patient records) and knowledge of the key features of the antiarrhythmic drugs (Table 1). Class I AADs have been shown to be safe and effective in patients with lone AF but contraindicate in patients with structural heart disease [29]. Hence, if no data on echocardiographic and other cardiac examination are available the patient should be referred to cardiologist for elective evaluation or rather a cardiologic consultation should be readily available in ED. Structured local instructions for treatment of AF patients and close collaboration with cardiologists play a key role in this process.

## Conclusions

Current survey is one of the largest studies evaluating AF treatment strategy and antiarrhythmic therapy in an emergency care setting. Our data indicate that rhythm control therapy is not understood and executed adequately in the ED. That is, in contrast to contemporary AF management guidelines a beta blocker was by far the most commonly used AAD and use of more effective drugs was rare also in symptomatic patients in rhythm control group.

## Abbreviations

AAD: antiarrhythmic drug
AF: atrial fibrillation
ED: emergency department
EHRA: European Heart Rhythm Association
ESC: European Society of Cardiology
FCS: Finnish Cardiac Society
OAC: oral anticoagulation therapy
